# SCHEPHERD: A modular, programmable, direct current platform to control cell behavior

**DOI:** 10.1126/sciadv.aed5802

**Published:** 2026-08-05

**Authors:** Yubin Lin, Jeremy S. Yodh, Celeste Rodriguez, Paul Kreymborg, Daniel J. Cohen

**Affiliations:** ^1^ Department of Electrical and Computer Engineering, Princeton University, Princeton, NJ 08544, USA.; ^2^ Omenn-Darling Bioengineering Institute, Princeton University, Princeton, NJ 08544, USA.; ^3^ Department of Mechanical and Aerospace Engineering, Princeton University, Princeton, NJ 08544, USA.; ^4^ Department of Molecular Biology, Princeton University, Princeton, NJ 08544, USA.

## Abstract

Spanning frogs, fish, and humans, direct current (dc) bioelectric cues play critical roles beyond neuromuscular function, such as modulating morphogenesis, immune response, and healing through electrotaxis—electrically directed cell migration. Harnessing this potential requires dedicated, versatile tools. However, standardized, accessible, and reproducible infrastructure capable of dc stimulation remains a challenge. We present SCHEPHERD: a universal, electrobioreactor integrating eight stimulation channels and modular inserts to enable most electrotaxis assays in one device (cells, monolayers, and 3D spheroids) while enabling powerful, expanded capabilities. SCHEPHERD revealed through parameter sweeps that dc fields act like a “steering wheel and gas pedal” for cell migration. We then used live confocal imaging to observe electrically reprogrammed F-actin dynamics. Last, our multipolar inserts generated complex spatial electrical patterns that reorganize engineered tissue dynamics. By substantially improving accessibility through modularity and an open-source, graphically programmed, stand-alone stimulator, we hope that SCHEPHERD can help broaden the community studying these important dc bioelectric phenomena.

## Teaser

SCHEPHERD enables complex electrical stimulation of cells and tissues to drive electrotaxis and electrobiological responses.

## INTRODUCTION

Bioelectricity plays a critical role beyond neuromuscular processes (*1*), including programming cell migration (*2*–*4*), regulating healing and disease (*2*, *5*–*10*), and directing morphogenetic processes (*5*, *11*–*14*). Unlike the familiar, rapid action potentials of the neuromuscular system, our focus here are slow-changing direct current [(dc) ion gradients producing fields of ∼1 V/cm in vivo] (*1*, *2*, *9*, *12*). These fields result from natural ion transport in epithelial tissues and can act on membrane-bound proteins to trigger electrotaxis—the directed migration of cells down an electric potential gradient—in nearly every tested model system, spanning slime molds (*15*), zebrafish (*5*), frogs (*4*, *12*), and mammalian models (*2*, *10*, *13*, *16*–*18*). The myriad roles of electrotaxis have sparked great interest in better stimulation tools to study the mechanisms of electrotaxis (*2*, *4*, *16*, *18*–*21*), control engineered tissues and organoids (*3*, *13*, *16*, *17*, *22*), alter development in vivo (*5*, *12*), and accelerate healing (*2*, *6*, *7*, *10*, *23*, *24*).

Unfortunately, while existing neuromuscular stimulation technology can safely deliver rapid pulses to trigger action potentials, the same hardware causes toxic redox reactions when used for sustained dc stimulation of the sort measured in vivo (*25*, *26*), necessitating development of bespoke devices that have been difficult to reproduce, control, scale, and integrate with complex biological systems. As a result, there is now no “standard” platform for electrotaxis, limiting progress through issues of reproducibility, throughput, modularity, and imaging (*6*, *27*, *28*). These formidable barriers-to-entry have not only reduced accessibility to the broader scientific community but also slowed our understanding of what appears to be a powerful biological phenomenon, and one goal here was to integrate best practices into a single platform.

We introduce a modular platform that both addresses many of these previous limitations while also revealing additional insights into the biology and biophysics of electrotaxis. SCHEPHERD (Spatiotemporal Cell Herding with Electrical Potentials for High-throughput Electrobiology with Reproducible Design) not only performs nearly all previously published live-imaging electrotaxis protocols in an integrated platform but also enables simpler setup and capabilities not possible with other approaches by incorporating programmable and modular electrical stimulation within a multiwell plate sample compatible with high-resolution multimodal imaging. We used high-throughput stimulation to sweep over different electrical field strengths and demonstrate that the directed migration of electrotaxis involves independent control of direction and speed, like a “steering wheel” and “gas pedal” used together. We next used high-resolution live fluorescence imaging to observe how electrical cues can reprogram F-actin stress fibers in real time, resulting in precise control of cellular maneuverability. Last, we showed how local electric field stimulation can trigger whole-tissue reorganization.

Our broader goal with SCHEPHERD as a platform is to provide a foundation to comprehensively serve and broaden the growing field of bioelectricity and to accelerate near-term work on wound healing, immune modulation, and tumor suppression. To enable this, SCHEPHERD is not only entirely open-source hardware and electronics but addresses a key accessibility concern for biology laboratories: We are committed to providing and supporting our custom electrical control boards and three-dimensional (3D) printed infrastructure, which should allow a higher level of standardization, reproducibility, ease of setup, and capabilities not possible in other systems.

## RESULTS

### SCHEPHERD is an integrated platform for reproducible and programmable stimulation

**Fig. 1.**
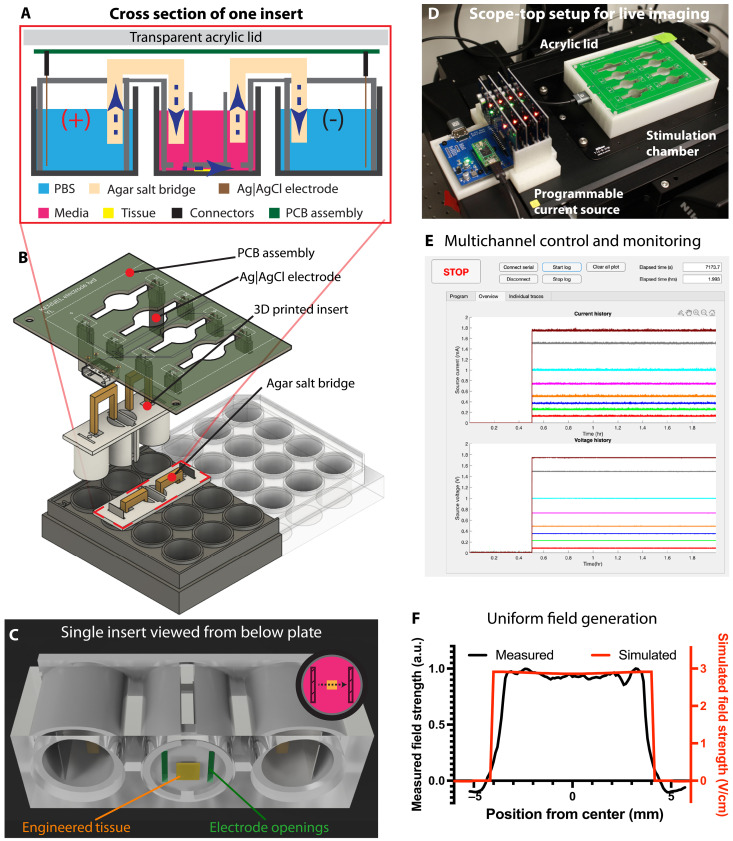
Device and platform overview. (**A**) Cross-sectional schematic of a single insert. A voltage difference is applied across an Ag (anode) and Ag/AgCl (cathode) electrode pair, generating an ionic current from PBS wells (blue), through agar salt bridges, into a media well (pink) containing a monolayer tissue (yellow). A transparent acrylic lid on top ensures confined area for CO_2_ perfusion. (**B**) 3D rendering of the multiwell insert. 3D printed inserts are slotted into a standard 24-well plate, agar bridges connect the PBS wells to the media well, and a PCB lid plate which contains the Ag and AgCl electrodes is placed on top of the 24-well plate. (**C**) 3D rendering of the bottom of a 3D printed insert showing how the field flows over a tissue. The insert has a lip around its perimeter used to maintain a ∼250-μm gap for current to flow through the two current openings. (**D**) SCHEPHERD on the microscope showing the enclosed 24-well plate with PCB lid plate assembly and the programmable current source. (**E**) Screenshot of the custom software used to control the eight independent stimulation channels. The specific current and voltage traces were used for the electric field sweep in Fig. 3. (**F**) The electric field in the *x* direction from a computer simulation (red) shows homogeneous stimulation across the well, which we validated with charge beads (black). a.u., arbitrary units.

What fundamentally sets dc bioelectricity apart from the more familiar ac (neuromuscular action potentials) is that it is a slow, steady process driven by sustained ion currents. However, slow and steady currents in an electrolyte will cause toxic redox reactions if not carefully managed, so all dc bioelectric systems follow a strategy similar to our approach in Fig. 1. First, a power supply drives current through nonredox electrodes [e.g., Ag/AgCl (*29*–*31*), conductive polymer (*32*–*37*), etc.]. These electrodes drive continuous ion flows carried through saline buffers and hydrogel salt bridges both couple to the living sample and filter out any harmful by-products. Current must then be concentrated over the biological sample as it is current density (current/cross section) that drives many dc biological processes (*24*, *27*, *38*–*41*), so samples are typically grown in microfluidic environments with small cross sections. A detailed discussion of design parameters has previously been presented (*3*), but the key design guideline is shown below in Eq. 1

$$\vec{E}=\frac{V}{d}\hat{x}=\vec{J}\rho =\frac{\vec{I}}{A}\rho$$ (1)

where $\vec{E}$ = electric field strength (volts per centimeter); *V* = voltage drop across the channel; d = length of culture channel, $\hat{x}$ = unit vector in the *x* direction; $\vec{J}$ = current density; $\vec{I}$ = current; *A =* cross-sectional area of the channel (square millimeters); and ρ = resistivity of the culture media (ohm per meter). Typical field strengths for dc cell biological experiments target O (1 V/cm). See prior work for more details (*3*, *16*, *18*).

Our approach is to encapsulate the entire stimulation process in a modular, 3D printed format that can be slotted into a variety of multiwell plates (adjusted accordingly) and essentially dropped directly on top of cultured cells, tissues, organoids, etc. that are already growing in the multiwell plate. Figure 1 (A to C) demonstrates how our approach can be implemented within a 24-well plate format. Here, the stimulation module occupies three contiguous wells within the plate, with the outer two wells containing paired Ag/AgCl electrodes immersed phosphate-buffered saline (PBS; brown and blue, respectively, in Fig. 1A), and the center well contains the sample to be stimulated immersed in cell culture media (yellow, Fig. 1A). We cast two agarose salt bridges in acrylic plastic molds so that they slot into and bridge the electrode wells to the culture well in the middle, providing a continuous electrical circuit and maintaining electrochemical separation (beige, Fig. 1A). Our monolithic, 3D printed insert is designed to settle into the plate under its own weight and naturally form the ceiling and walls of a precise tunnel with a height of 250 μm (adjustable) around the cultured sample (yellow, Fig. 1A). Openings in the ceiling of this central insert allow ionic currents to flow through this effective microfluidic channel and to stimulate the sample with high current density. 3D printing allows slight modifications made to fit inserts to different 24-well plates (see Methods). Figure 1B shows how multiple modules can slot into a multiwell plate, while Fig. 1C shows a view from below a mounted insert where a square cultured tissue is stimulated between two parallel electrode openings in the ceiling. The direct integration into multiwell plates allows us to leverage existing, standardized culture systems and support most forms of inverted, live-cell imaging. The field uniformity is verified with electrophoresis from the charged fluorescent beads (Fig. 1F), and the result agrees with the computer simulation; see Methods for details.

Up to eight of these three-well modules can be loaded simultaneously in a 24-well plate (see Fig. 1D), which we made far more manageable by introducing a custom printed circuit board (PCB) “lid” that incorporates all wiring, automatically aligns the Ag/AgCl electrode films in each well, and provides space for salt bridges and microscopy imaging [green PCB in Fig. 1 (B and D)]. This customized lid also allows the platform to more easily integrate into live microscopy setups, and the concept can be generalized for a variety of multiwell plate formats. All modules are fully programmed, monitored, and driven through a single cable that connects the PCB lid directly to our custom power supply.

The final element of any dc electrobioreactor is the power supply, which ultimately limits the kind of stimulation that is possible. In practice, most of the dc stimulation studies rely on repurposed laboratory power supplies (*38*, *42*–*47*) (often lacking proper safeguards and resolution) or professional current sources (*17*, *24*, *40*, *48*) (costing thousands of dollars). While custom solutions exist (*41*, *49*), no extant system (including our own prior work) effectively combines high-throughput, programmable, safe, and fully logged dc stimulation as the hardware does not exist. Moreover, the wide variation in power supply setups across studies can make it difficult to replicate prior studies or even set up a safe stimulation system. We solved this issue by designing and integrating a custom, eight-channel current source with independent programming and data logging for each channel (Fig. 1E and figs. S1 and S2). This system was built from the ground up for dc stimulation during live microscopy. Power delivery is handled through precision current sources for constant current control, which is far more reproducible for dc electrobiological use and also compensates for the interfacial resistances between electrodes and electrolytes to guarantee a stable electric field across the sample throughout an experiment. The miniature format of our inserts allows for low operating voltages (up to 20 V), thereby improving safety by reducing shock hazards (*45*, *50*), and our single-cable connection between this custom power supply and the stimulation plate eliminates the wire and salt bridge tangle typical of prior electrotaxis systems (including our own). Now, a 1-mA driving current will produce ∼2 V/cm within a stimulation well, and this can be independently adjusted for up to eight wells. This entire interface is a fraction the cost of commercial solutions while enabling unique functions, and a complete bill of materials and design guides are provided in the Supplementary Materials. Figure 1D illustrates the complete system setup that includes the stimulation controller, the stage top live imaging SCHEPHERD, and a surrounding enclosure for CO_2_ delivery and temperature control.

### Validation of the dc stimulation for common experimental approaches

**Fig. 2.**
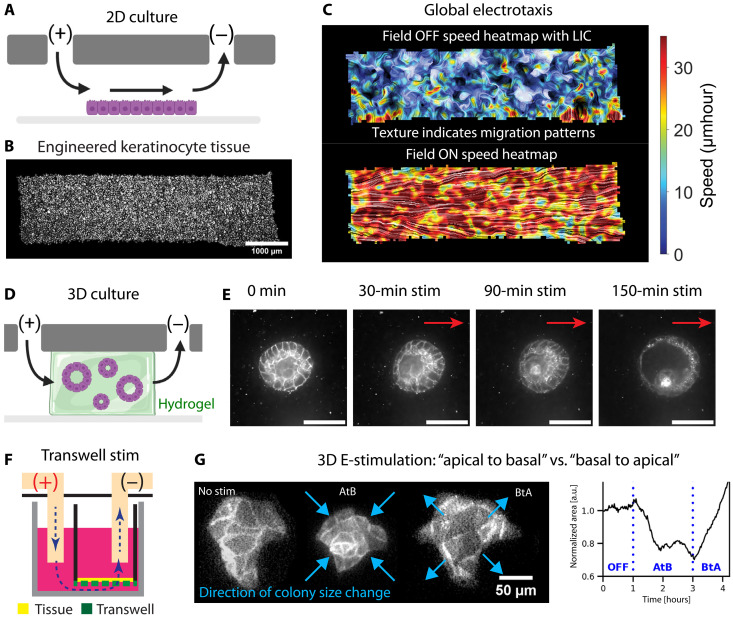
Reproducing the canon of electrotaxis results with the SCHEPERD assembly. (**A**) Schematic of homogeneous current flow over a tissue monolayer. (**B**) A micrograph of a 6 mm–by–1.5 mm monolayer tissue of primary mouse keratinocytes (see Methods). Scale bar, 1000 μm. (**C**) Migration flow pattern (see Methods) overlaid on a speed heatmap without electric stimulation (top) and with electric stimulation (bottom) shows a uniform response to the field. Color corresponds to speed magnitude, and texture shows streamlines. LIC, Line Integral Convolution. (**D**) Schematic of electrically stimulating 3D kidney cysts embedded in a hydrogel (see Methods). (**E**) Confocal slice over time of electro-inflation of the cyst. Scale bars, 50 μm. (**F**) Schematic of transverse (vertically oriented) stimulation through a transwell showing apical-to-basal (AtB) and basal-to-apical (BtA) stimulation. (**G**) Micrographs of an MDCK colony which contracts with AtB stimulation and then expands with BtA stimulation. The normalized area dynamics are plotted showing that AtB induces contraction and BtA induces expansion of the island.

We next validated an individual stimulation module (the three-well insert) against challenging cases from the literature involving unidirectional electrical stimulation that would normally require different setups. We first targeted generating a uniform electric field across an engineered tissue by designing an insert with parallel electrode openings in the ceiling to produce a uniform electric field (Fig. 2A). We tested against large-scale collective electrotaxis (*3*, *13*, *16*, *51*) as this is relevant for a range of studies involving healing and development (movie S1). Here, a whole tissue is stimulated with a uniform electric field to drive cell migration from anode to cathode. Collective electrotaxis is an important validation case both because of the scale of the sample (e.g., centimeters) and the need for the electric field to be uniform over that large area. To demonstrate this, we engineered a precise epithelial monolayer of primary undifferentiated mouse keratinocytes 1.5 mm by 6 mm in size (see Methods) and drove its electrotaxis with a 3-V/cm field (Fig. 2B). They are a strongly electrotactic cell type and serve as biological readout of field uniformity. As shown in Fig. 2C, we observed tissue-scale collective migration, demonstrating both the expected increase in speed with electrotaxis and highly directional motion (aligned parallel to the applied electric field). SCHEPHERD mainly uses fluorescence imaging to maximize its flexibility. However, with minimal modification, the concept of SCHEPHERD was easily adapted for transmitted light imaging. Figure S5 shows the schematic of the insert, where two pieces of acrylic provide imaging window for the transmitted light path. We further tested this with MDCK (Madin-Darby canine kidney) tissue under 3-V/cm stimulation in movie S2. MDCK has stronger cell-cell adhesions and exhibits more collectivity during electrotaxis, making it a more challenging system to control.

Our second test case was 3D dc stimulation of “epithelioids”—hollow, cyst-like structures of epithelial cells grown in hydrogels that have previously been shown to both inflate (“electroinflation”) and break symmetry during dc stimulation (*13*). This type of stimulation was previously performed with confocal imaging and continuous media perfusion in a relatively complex device (our former design). To accommodate the working distance required for confocal imaging, we grew the tissues in a hydrogel within the well of a glass-bottomed 24-well plate and then gently slotted the stimulation insert into the well to form the stimulation channel around the tissues (Fig. 2D). Subsequent electrical stimulation recapitulated the previous data, showing rapid inflation and symmetry breaking (thinning on the anode side and thickening on the cathode side; Fig. 2E and movie S3).

Last, we tested transwell stimulation—an advanced culture mode where a semipermeable culture membrane separates upper and lower media chambers that is used for electrical stimulation in complex culture environments (*52*, *53*) and for transmigration experiments with immune cells (*54*, *55*). Here, we modified our printed insert to accommodate a commercial transwell platform (see Methods) and allow us to direct an electric field perpendicular to cultured cells (Fig. 2F). Previous work with epithelial stimulation has shown that stimulating in the AtB (apical-to-basal) direction causes cells to contract, while stimulating BtA (basal-to-apical) causes cells to expand (*56*). Our data replicate this, demonstrating strong contraction and expansion of islands of epithelial cells over hours of AtB/BtA cycles (Fig. 2G and movies S4 and S5). Together, these three demonstrations show how SCHEPHERD can recapitulate prior results from a wide range of different culture and stimulation conditions all within a single platform, emphasizing that one platform can handle most of the different experimental needs.

### Electrotaxis separately regulates speed and direction via a “steering wheel and gas pedal” motif

**Fig. 3.**
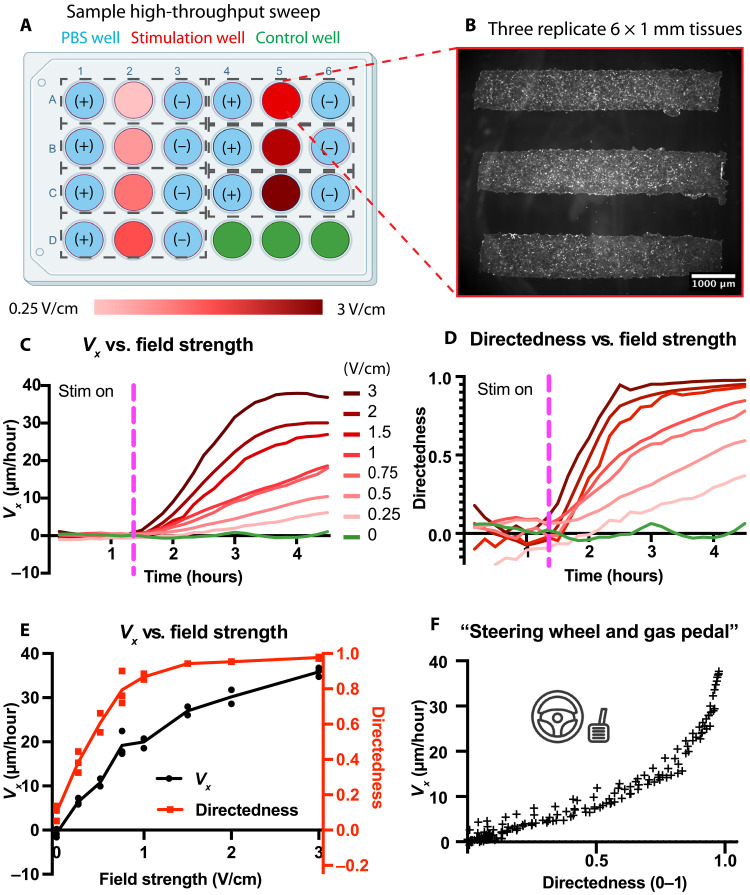
SCHEPERD device massively increases experimental throughput. (**A**) A 24-well schematic of an electric field amplitude sweep. Each triplet of wells contains an independent stimulation module (as in Fig. 1A) with an anode (+) and cathode (−) pair flanking a stimulation well. With this assembly, a 24-well plate contains up to eight stimulation triplets, allowing for eight independent experimental conditions; here, three wells were devoted to negative controls. In the schematic, color corresponds to field strength. (**B**) A micrograph of one well with three 6 mm–by–1 mm MDCK tissues. Scale bar, 1000 μm. (**C**) The dynamics of the *x* component of the velocity for the stimulation sweep show that increased field strength results in increased *x* velocity. (**D**) Dynamics of the directedness (measured with respect to the applied electric field) as a function of field strength that the directedness saturates faster with stronger fields. (**E**) Transfer function of the *x* component of the velocity and the directedness as a function of electric field strength. Data points were taken 3 hours into stimulation. (**F**) Speed plotted as a function of directedness showing at least two distinct regimes of behavior. For relatively low directedness (<∼0.8), speed increases linearly with directedness. At higher values of directedness (>0.8), the speed scales super-linearly with directedness. These data emphasize our steering wheel and gas pedal framing of electrotaxis.

We next evaluated capabilities uniquely enabled by SCHEPHERD. A key challenge in dc bioelectricity is relating the stimulation input for a given model system to specific output (e.g., migration speed) given the wide range of possible electrical inputs. While resistive ladder strategies have been demonstrated in the past for single cells (*41*, *44*), high-throughput stimulation of engineered tissues is much more difficult, and prior work in this space typically sticks to previously established field strengths (*3*, *16*, *18*, *51*). Here, we tested and demonstrated the high-throughput capabilities of SCHEPHERD to sweep eight different field strengths spanning 0 to 3 V/cm in a single experiment using our multichannel, 24-well plate from Fig. 1. The final three wells were used as negative controls (0 V/cm). Here, the plate was configured as shown in Fig. 3A, and to further emphasize throughput, we engineered three replicate tissues per stimulation well (Fig. 3B and movie S6). This allowed us to capture a complete sweep of tissue responses within a single experiment with multiple replicates per condition. Figure 3 (C and D) shows the dynamics of cell migration speed or directionality (0 = undirected motion, 1 = fully aligned with the electric field vector). The high throughput of this sweep allowed us to construct a “transfer function” plot (Fig. 3E) cleanly mapping the directionality and speed of the bulk of the tissue to a given electrical input. In this case, each tissue would count as one biological replicate (thus three in every stimulation group), and each well counts as a technical replicate. Users are free to arrange different combinations of stimulation settings to achieve desired sufficiency of data. However, regardless of the definition of replicates, it must also be noted that performing this kind of sweep with any extant system would take at least 1 month of microscope time and sample preparation to serially run the experiments and replicates, whereas we were able to complete all replicate sweeps in a single day of back-to-back imaging.

However, these finely mapped data raised another question—why do cells drastically speed up during electrotaxis, as shown in our transfer function (Fig. 3F) and as noted in prior studies (*3*, *18*, *51*)? One hypothesis in the field is that electrical stimulation causes joule heating of the media around the cells and that migration scales with temperature (*4*). We ruled that out here as part of our validation of SCHEPHERD where we measured temperature in the tissue culture channel during stimulation and observed no obvious temperature shifts (fig. S4).

A competing hypothesis, known as the “compass model” of electrotaxis (*51*, *57*), is that speed increases because directedness is increasing. Essentially, as the direction of motion of individuals in a crowd becomes more uniform, there should be less frequent neighbor-neighbor collisions, which increases movement efficiency and allows cells to reach higher speeds. If true, this hypothesis suggests that speed should saturate when directedness saturates, i.e., when directedness reaches ∼1, speed should plateau. A direct way to test this argument is to correlate speed with directionality for all data points from our field sweep (see Fig. 3F), where each data point is described by a speed and a directedness. Here, we see that speed and directedness are linearly related for low values of directedness (<0.8); but as directedness reaches its maximum of 0.8 to 1, speed markedly increases with a larger characteristic slope (although we note that this behavior is nonlinear). This finding proves that the compass model of electrotaxis is incomplete. We instead suggest thinking of tissue-scale electrotaxis as a “steering wheel and gas pedal” process. The direction of the electric field sets the migration direction (steering wheel), and the magnitude of the electric field sets speed (gas pedal). At low fields (*E* < 1 V/cm), migration speed increases from both the steering wheel and gas pedal, but at high fields (*E* > 1 V/cm), when steering can no longer boost efficiency, migration speed still grows via the gas pedal.

### F-actin stress fiber networks pivot in response to dynamic electric fields

Controlling mammalian cell migration ultimately requires controlling the actomyosin cytoskeleton—the force generating machinery of the cell—but how this occurs in electrotaxis is not well understood. This matters because the cytoskeletal response sets what we can and cannot do with electrotactic control. Prior work has clearly established localized actin build-up at the leading edge of electrotaxing cells across many fast moving cell types and organisms including *Dictyostelium discoideum* (*58*–*60*) and mammalian cells (e.g., neutrophil-like cells) (*61*). These studies connect classical cell polarity signaling [e.g., phosphatidylinositol 3-kinase (PI3K)/Phosphatase and Tensin homolog (PTEN)] and cytoskeletal regulation to rapidly localizing actin signals (*62*). However, less is known about how slower migrating cells that use the more mesenchymal crawling mode of migration (e.g., epithelia, endothelia, and fibroblasts) reorganize their cytoskeletons, where a key driver of migration is the contractile network of F-actin stress fibers (*62*, *63*). Much of the data relate to fixed cells, losing the temporal resolution but showing long-term alignment of stress fibers perpendicular to the electric field (*62*, *64*–*68*).

Here, we asked a new question: How does the cell dynamically structure this network of contractile fibers? Two hypotheses would be that: (i) Cells disassemble the existing network and reassemble it in response to the field; or (ii) cells reconfigure the existing network. Assessing this requires precise electric field biaxial direction control, high resolution, and live fluorescence imaging on mammalian cell models in one experiment. We address this problem here with SCHEPHERD inserts for 2D, programmable E-field vectors in combination with standard glass-bottomed petri dishes.

**Fig. 4.**
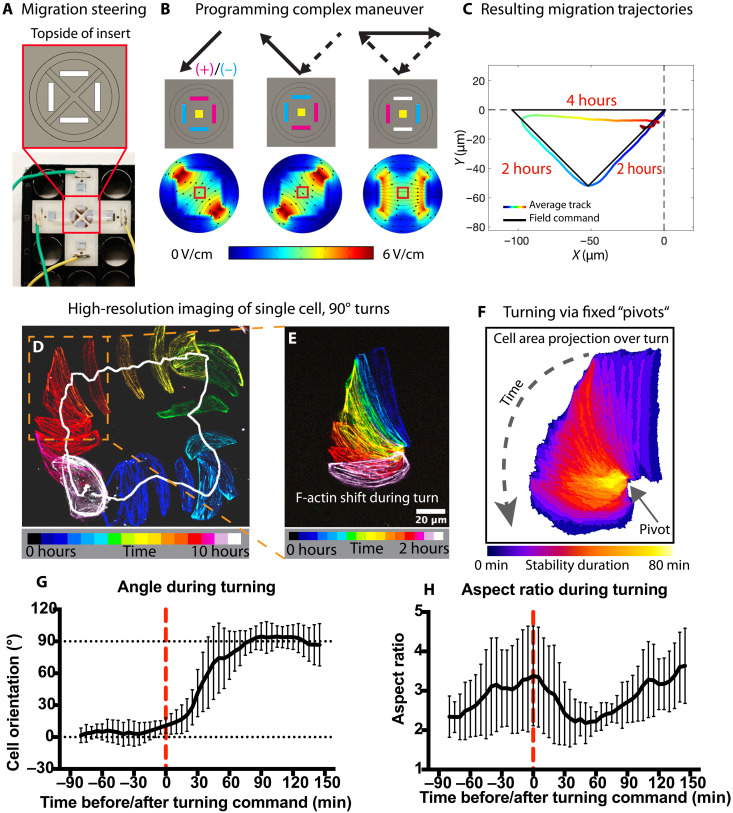
Electrically steering single-cell migration and cytoskeletal dynamics in 2D. (**A**) Schematic of the top of insert (top) and the physical experimental setup (bottom) for *XY* electric stimulation. (**B**) Top row: programmed command vectors. Middle row: anode and cathode addressing schematics. Bottom row: 2D COMSOL simulations showing the field strength and direction. (**C**) Averaged trajectories (*N* = 200) over a population of primary mouse keratinocytes in time subject to the 2D triangular migration command from (B). (**D**) High-magnification imaging of the cytoskeleton where color corresponds to time, the cells are programmed into a square trajectory. The white solid line shows the trajectory of the centroid of the cell. (**E**) Cell rotating during a right turn color corresponding to time in 15-min interval. (**F**) Overlap outline of (E), indicating the swinging motion from a fixed pivot. Color indicates the duration of the overlap area in a turning event. (**G**) Cell orientation under turning command (*N* = 9). The red dashed line indicates field direction changes. (**H**) Cell aspect ratio changes under turning command.

We implemented full 2D electrical control by adding a second axis of electrodes to our SCHEPHERD insert (see Fig. 4A) that we independently control, meaning that we can set any arbitrary in-plane electric field direction by shifting the relative weights of how strongly biased the electrodes are and which are “+” or “−” as shown in the cartoon in Fig. 4 (A and B). This approach is more complete in imaging and stimulation capabilities and critically more accessible than our prior work, the SCHEEPDOG platform where perfusion, low-magnification, and low-throughput are constraints (*16*). To ensure this approach worked, we first demonstrated programming a “triangular” maneuver (Fig. 4B) into an array of cultured primary mouse skin cells and plotted the average of their tracked trajectories over an 8-hour period (Fig. 4C; see Methods and movie S7).

We next switched to high-resolution [40×/1.3 numerical aperture (NA)] live imaging of the fluorescently labeled F-actin cytoskeleton in keratinocytes and programmed multiple 90° turns to generate a “square” trajectory (see Fig. 4D and movie S8). During the straight segments, we observed cells fanning perpendicular to the applied field direction [reported in prior transmitted light studies (*4*)] with long actin stress fibers forming a lamellipodium at the leading edge (Fig. 4D). During 90° turns, however, we observed cells to “swing” the lamellipodium around a fixed, stable pivot to complete the turn rather than depolarizing and repolarizing a new lamellipodium (Fig. 4, E and F). This turning process involved a smooth rotation of the cell long axis (Fig. 4G) and always exhibited a sharp decrease in a cell aspect ratio during the turn itself (Fig. 4H), which suggests a deformation of the lamellipodium rather than reassembly and warrants further study to understand how the local electric field can drive this. From the perspective of filament bundles and liquid crystals, this pivoting process, wherein stress fibers maintain their locally aligned orientation as the cell turns, is likely energetically favorable. During the initial stimulation, the electric field induces actin stress fiber reorientation from an isotropic state to a lower energy nematic state. Rearranging or distorting this fiber orientation field will cost energy, and therefore as the cell pivots, maintaining its cytoskeletal orientation field is favorable to disassembling or rebuilding the structure (*69*).

### Spatial electric field stimulation patterns induce whole tissue deformation

In vivo data demonstrate that naturally occurring dc fields tend to have spatiotemporal patterns, as might be expected from the complex shapes, sizes, and functions of living tissues (*5*, *12*, *70*). However, nearly all electrotaxis studies have focused on global stimulation with uniform electric fields (including everything shown so far in this work), in large part due to experimental constraints. SCHEPHERD’s ceiling-based electrode system offers a distinct approach to locally shaping the electric field. Electric fields have an intrinsic direction from (+) to (−), so patterning where the anodes (+) and cathodes (−) are in the ceiling relative to a tissue should create regions of expansion and contraction.

**Fig. 5.**
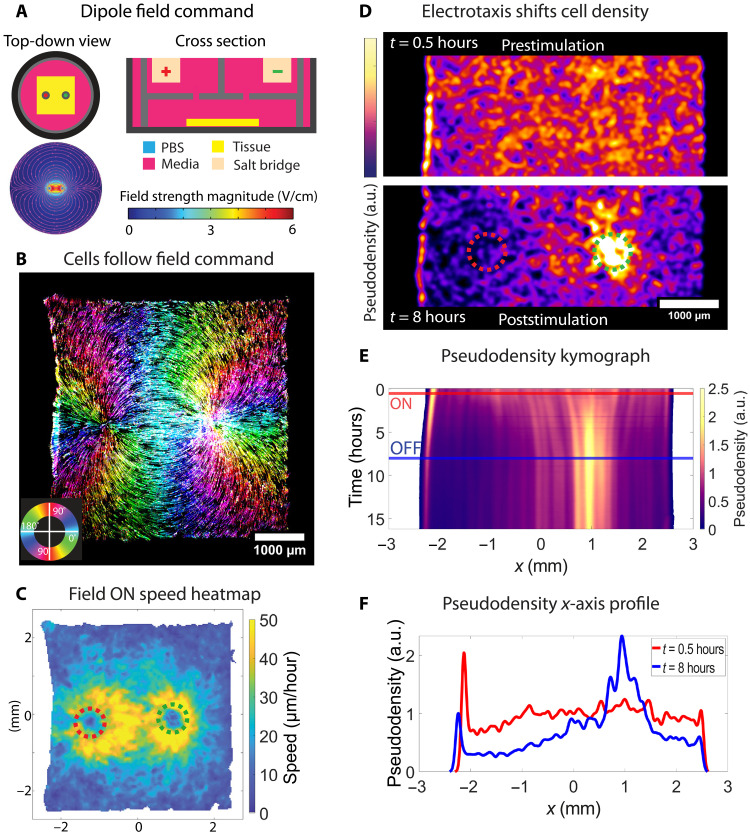
Spatial electric fields generate complex, designable migration patterns, cell density variations, and tissue changes in living tissues. (**A**) Left: Top-down view of the anode and cathode geometry in the insert ceiling to generate a dipolar field and a 2D COMSOL simulation of the field. Right: Cross-sectional view of the dipole inserts. Note that the openings of the field are between the tissues that enable locally applied field. Schematics drawn not to scale. (**B**) Flows and orientations of cell trajectories show that the cells follow the field lines produced by the insert. Color corresponds to orientation. Scale bar, 1000 μm. (**C**) Speed heatmap averaged over hours 1 to 2 or stimulation showing dipolar migration response. (**D**) Plot of the pseudodensity of nuclei when stimulation is turned on at *t* = 0.5 hours (top) and immediately after stimulation is shut off at *t* = 8 hours (bottom). The pseudodensity maps show build-up in cell density at the cathode and decreased density at anode. Color corresponds to pseudodensity. (**E**) Kymograph of the pseudodensity. Horizontal red and blue lines correspond to when the field turns on and off, respectively. The density flows toward the cathode in time, located at *x* ∼ 1 mm. The bright region around *x* ∼ −2.5 is an artifact of edge damage. (**F**) Two line profiles of pseudodensity taken when stimulation (stim) is turned on (*t* = 0.5 hours) and right after stim is turned off (*t* = 8 hours) which show that density increases at the cathodal side and decreases (pseudodensity < 1) at the anodal side. These line profiles are taken at times which correspond to the horizontal lines in (E).

To demonstrate this, we created an insert with a ceiling containing two independent, small electrode openings aligned over small regions of a much larger tissue (Fig. 5A). Activating these (Fig. 5A) could induce dipolar electric field pattern and could result in complex local migration dynamics within a 5 mm–by–5 mm tissue sheet of primary skin cells, and our experimental data validated this. Figure 5B and movie S9 reveal a complex pattern of converging and diverging cell migratory flows within the tissue, with the rainbow colors indicating the local migration axes orientations. Beyond simply controlling direction and speed (Fig. 5C), these complex patterns have implications for local cell density (Fig. 5, D and E) and even overall tissue shape, as the patterned fields ultimately sculpt the global shape and size of the tissue; the left and right boundaries of the kymograph in Fig. 5E show the tissue expanding and contracting respectively. While stimulation is on, pseudodensity accumulates under the cathode. This is evident in pseudodensity heatmaps shown in Fig. 5D and the pseudodensity kymograph and line profiles in Fig. 5 (E and F). The ability to pattern both expansion and contraction with a single input sets electrical stimulation apart from other patterning approaches such as optogenetic regulation of collective cell migration (*71*).

## DISCUSSION

Bioelectricity beyond the brain is implicated in countless critical processes—immune activity (*11*, *72*, *73*), wound healing (*6*, *23*, *70*), development (*74*, *75*), transplant viability (*76*, *77*), and cancer treatment (*78*–*81*)—that purpose-built tools are needed to fully capture the potential and push the field forward. While SCHEPHERD is not perfect, we hope it is a positive step and solid foundation toward advancing the field both by making these studies more accessible to a broader, interdisciplinary community and by enabling additional capabilities that expand the envelope of what we can do with bioelectric stimuli.

That said, what are some remaining limitations of the SCHEPHERD platform and how might they be addressed? The most obvious is that we have now only tested SCHEPHERD using a cage incubator built around a time-lapse microscope, which maintains physiological temperatures as this is the instrumentation we had available. Additionally, cage-style incubation does not restrict how much instrumentation or space a sample or apparatus can occupy. However, the modularity of SCHEPHERD is such that it should be readily adaptable to small-footprint, stage-top incubators for live imaging through the use of 3D printed or machined adapters. In addition, there is still a small amount of electronics assembly needed. In terms of the stimulation itself, SCHEPHERD’s programmable power supply was deliberately built to favor the slow, dc fields measured in vivo rather than ac stimulation that is more common for neuromuscular stimulation or tumor-treating fields that disrupts mitosis. However, the SCHEPHERD culture system itself can be directly integrated with any commercially available stimulators if needed, such as high-frequency ac units. Perhaps the most frustrating part of SCHEPHERD reflects a challenge in the whole field of dc bioelectricity—how do we safely generate dc fields without toxic redox? While the Ag/AgCl electrodes used in SCHEPHERD remain the “gold standard” for biocompatible stimulation (*29*–*31*), this is wasteful of silver, and the total charge that can be delivered is limited as the AgCl layer is sacrificial and eventually depletes. However, exciting work is taking place to develop next-generation biocompatible electrodes for dc stimulation through the use of conductive polymers and pseudocapacitors (*32*–*37*). As these come online, they can be directly swapped into the SCHEPHERD architecture and use the platform as a reliable test bench to evaluate charge capacity or biocompatibility against AgCl or other electrode technologies. Last, we encourage the community to adapt SCHEPHERD for use with single petri dishes, as not all experiments require multiwell plates.

Getting started with electrotaxis and dc stimulation studies can be a barrier to entry given the various factors that must be balanced, and a simple starting place can be helpful. A variety of entry points exist when multiwell plates or high-resolution imaging is not required, spanning vacuum grease slides (*2*), double-sided tape flow chambers (*3*, *44*), daisy-chained wells (*22*), transwells (*21*, *73*), and many more. Moreover, these platforms can be used with the fully open-source, programmable, multichannel, and data-logging aspects of the stand-alone SCHEPHERD power supply to improve electrode reliability, data logging, control, and throughput. Ultimately, researchers are encouraged to mix and match best practices and components and continue to advance the field.

All of that said, we are genuinely excited to release and share SCHEPHERD as it now represents the most reliable, user-friendly, versatile, and high-throughput platform we have tested and will hopefully enable far more researchers to enter this field. It supports from single cell to 3D tissue models, allows up to eight simultaneous stimulation patterns, and enables real-time, shaped control of the electric field. Moreover, we are committed to making SCHEPHERD accessible (see GitHub link in “Data, code, and materials availability”) and supporting its further development both through direct interactions and by sharing all the necessary plans, schematics, circuitry, and software to run SCHEPHERD and start herding cells.

## METHODS

### Device manufacturing

Each stimulation insert was 3D printed using white polylactic acid (PLA) filament (Polylite PLA natural, Polymaker) using a 3D printer equipped with 0.4-mm nozzle (X1C, Bambu Lab) and with a slicer setting of 80% infill, 0.08-mm layer height, and support. A polyethylene terephthalate glycol filament [Polylite PETG (Polyethylene Terephthalate Glycol) gray, Polymaker] was used for interfacing the support and the main structure for ease of removal. The high temperature (220°C) during extrusion ensures sterility inside the insert (*82*). After printing, the supports were removed using a tweezer, and then each insert was dialyzed in 500 ml of sterile deionized (DI) water overnight. Incubator enclosure was also 3D printed with a white PLA filament (Polylite PLA natural, Polymaker), and the enclosure lid was made from laser cutting a 2-mm-thick acrylic sheet (Makerstock) (VLS 2.7, Universal Laser System).

Agar salt bridges were prepared mixing agarose (20-102, Apex Bioresearch) and Dulbecco’s phosphate-buffered saline (DPBS) (D8537, Sigma-Aldrich) at 4% (w/v) on a hot plate set to 130°C. Once fully melted, the molten agar was cast into a mold made from laser cutting a 3-mm-thick acrylic sheet on a glass slide. After agar was solidified, a sterile safety razor was used to trim the excess agar, and then the glass slide was removed to pop the molded salt bridges with a tweezer into a dish filled with DPBS to keep agar bridges hydrated. Bridges with bubbles were discarded.

Ag|AgCl electrodes were prepared by cutting silver foil (AA11440GW, Thermo Fisher Scientific) into 8 mm–by–23 mm strips. Each electrode was plated in 1 M KCl solution (P9541-1KG, MilliporeSigma) for 4 hours with a current of 3 mA and a titanium wire cathode (00362-G1, Alfa Aesar). The coated portion should be around 8 mm by 15mm. Then, the coated Ag|AgCl cathode and Ag anode were or inserted into the PCB lid or soldered to 24 AWG wires (only for biaxial stimulation). The electrodes had a charge capacity of 12 mAh, and no more than 8 mAh of charge was used in each experiment to prevent faradaic reaction due to depletion of AgCl at the cathode.

### Joule heating measurement

Temperature rise due to joule heating was measured from placing a T-type thermocouple (5TC-KK-T-30-72, Omega) at the bottom of the well while running stimulation (see fig. S4). Various stimulation strengths up to twice the normal operating current were applied. The temperature rise readout (54 II B, Fluke) was less than 1°C; see fig. S4 for temperature deviation.

### pH stability measurement

Media from the eight stimulation wells was measured after a stimulation delivery of 3 V/cm for 3 hours using a portable pH meter (pH-33, Horiba). The stimulation well average was 7.29 (±0.12 SD). pH gradient across the channel was measured by sampling 200 μl of media at each end of the four stimulation wells using a gel loading tip. The results do not show significant pH gradient with *P* = 0.671.

### Field uniformity verification through simulation and electrophoresis

Insert geometry was modeled in a finite element software (COMSOL 5.6) using a material conductivity of 1.6 S/m and a relative permittivity of 80. It shows less than 10% variation in field a 6 mm–by–6 mm center, which aligns with many of similar devices’ design goals (*16*). Actual field strength was measured by inserting two Ag/AgCl-coated probes into the bottom of the insert. The measured voltage agrees with the COMSOL simulation. However, to further validate the result of the stimulation, we inject charged fluorescent beads into the stimulation well and measure its speed due to electrophoresis. Fluorescently labeled charged beads (F-8765, Thermo Fisher Scientific) was diluted 1:1000 in DPBS and then added 1.5 ml to the stimulation well. After device assembly as shown before, the setup was imaged using a confocal microscope (NL5, Confocal NL) with a 10× 0.3 NA objective in a fluorescein isothiocyanate (FITC) channel with a frame rate of 5 frames per second on a scientific Complementary Metal–Oxide–Semiconductor (sCMOS) camera (Fusion BT, Hamamatsu). Electrophoretic force on charged fluorescent bead reaches equilibrium with the Stokes drag of the fluid. Therefore, bead speed is linearly proportional to the field strength. Particle image velocimetry (PIV) analysis was then performed on the captured images.

### Cell culture

Primary mouse keratinocytes were provided by the Devenport Laboratory at Princeton University and cultured in E-medium (*83*) supplemented with 15% serum (S11550, Atlanta Biologicals) and 50 μM calcium in the form of calcium chloride. Wild-type MDCK-II cells (courtesy of the Nelson Laboratory, Stanford University) were cultured in Dulbecco’s modified Eagle’s medium (DMEM) (D5523-10L, Sigma-Aldrich) with sodium bicarbonate (1 g/liter), 10% fetal bovine serum (S11550, Atlanta Biologicals), and 1% penicillin-streptomycin (15140-122, Gibco). All cells were maintained at 37°C under 5% CO_2_ and 95% relative humidity. Cells were split before reaching 70% of confluence for maintenance culture and were discarded after passage 30.

### Generation of TMEM154-eGFP MDCK cell line

The generation and integration of the fluorescent Transmembrane protein 154 (TMEM154)–enhanced green fluorescent protein (eGFP) fusion construct used in this study have been previously described (*21*). In short, the construct was synthesized (GenScript) and cloned into a PiggyBAC transposon backbone and then stably integrated into MDCK-II cells via Lipofectamine LTX transfection (15338100, Thermo Fisher Scientific), followed by single-cell sorting (MA-900, Sony Biotechnology Inc.) and clonal expansion. Clones were screened for stable eGFP expression and proper membrane localization by flow cytometry (Invitrogen Attune NxT) and fluorescence microscopy.

### Tissue patterning and labeling

Tissue patterning was similar to previously published protocols (*16*, *18*, *84*). For tissue-wide imaging, 24-well plastic-bottom plates (EW-01927-74, Cole-Parmer) were used, and for imaging with immersion objective, 24-well glass-bottom plates (P24-1.5H-N, CellVis) were used for working distance compliance. Inserts were modified for the different plates and are included in the repository. Plates were coated with human plasma fibronectin purified protein (FC-010, MilliporeSigma) for keratinocyte experiment or collagen IV (C5533-5MG, Sigma-Aldrich) for MDCK experiment. Each well was coated with 90 μl of fibronectin (50 μg/ml) or collagen under a 15-mm circular glass coverslip (CLS-1760-015, Chemglass) for 30 min under 37°C and then was washed three times with DI water. After allowing the dish to air dry, stencils were cut from 250-μm-thick silicone (HT-6240, Stockwell Elastomers) using a Silhouette Cameo vinyl cutter (Silhouette, USA) and transferred to the fibronectin-coated wells. One hundred twenty-five microliters of media was added around the edge of each well to keep it humidified during seeding and attachment.

Keratinocytes were concentrated at a concentration of 2.25 × 10^6^ cells/ml and then pipetted into the stencil for ∼1000 cells/mm^2^, and care was taken not to scratch the fibronectin-coated substrate. Then, the plate was incubated for 4 hours, allowing keratinocytes to attach to the substrate. After attachment, 500 μl of media is added in each well, flooding the tissue. The stencils were lifted after 18 hours, which allows cells to form a confluent monolayer before stimulation. Cellbrite red (30023, Biotium) was added 5 μl/ml of media, and each tissue is stained with 500 μl of staining media for 30 min, and then replaced with normal culture media for imaging. For nuclear tracking, Hoechst 33342 (D1306, Thermo Fisher Scientific) was used 1:1000 for 30 min. For actin labeling, cells were stained with SPY555-Actin (SC202, Spirochrome) 1:1000 for 1 hour and were imaged with the probe in media. The density for single-cell imaging is ∼50 cells/mm^2^

MDCK cyst culture was described previously (*13*). In short, MDCK cells were concentrated at a concentration of 2.25 × 10^5^ cells/ml in DMEM solution. Geltrex matrix (A1413201, Gibco) was mixed with the media at a 1:1 ratio. A total of 3.4 μl of the resulting mixture was deposited onto the opening of the poly(dimethylsiloxane) stencil. After solidifying for 30 min, 2.9 μl of the mixture was deposited onto the first layer. The gel was then incubated for 1 hour before flooding with DMEM. The cysts were cultured for 96 hours and were imaged using Memglow 640 (MG04-10, Cytoskeleton Inc.). The sample was stained for 30 min using 100 nM concentration.

For transwell stimulations, approximately 86,000 TMEM154-eGFP MDCK is seeded to a 0.4-μm pore size transwell membrane insert (353180, Corning). The samples were then incubated for 18 hours before the experiment. Samples were imaged in an FITC channel.

### Device assembly

All components except salt bridges and inserts were sterilized by ethanol and then air dried in tissue culture hood. Agar salt bridges were sterilized by exposure to ultraviolet (UV) radiation for 5 min. The inserts were placed in sterile DI water immediately after printing and were also UV sterilized. The 24-well plate is placed onto a hot plate (946C+, Soiiw) set at 50°C to keep cells at 37°C during assembly and reduce focus drift due to thermal expansion in imaging. Two milliliters of PBS was added for each well that did not contain tissues, and 1.5 ml of media was added to each tissue well. Next, the inserts were carefully dropped into the wells. Then, the plate was inspected from the bottom for any trapped bubbles, which may compromise field uniformity.

Next, agar salt bridges were inserted after drying the excess PBS onto a piece of Kimwipe (06-666, Kimberly-Clark Professional). The PCB lid was then dropped down with the electrodes aligned with each insert. For biaxial stimulation, electrodes were inserted into the slits on the 3D printed insert, and the wires were fixed using adhesive mounting putty (2600205, Loctite) for strain relief. The assembled platform was ready to transfer to the microscope while maintaining a sterile condition for the samples. Any unused wells were filled with PBS to provide extra tolerance for evaporation. The total assembly process is around 10 min on the hot plate, minimizing the evaporation due to heating. The assembly can also take place in room temperature and then equilibrated inside a humidified, heated incubator before mounting on the microscope. Since media conductivity will vary with temperature, samples were then equilibrated to 37°C before stimulation started.

### Stimulation delivery and instrumentation

An eight-channel programmable current source was designed to minimize device footprint and provide easy stimulation control. It is capable of sourcing or sinking up to 20 mA or 20 V per channel. It can also support constant voltage mode and four-wire kelvin feedback with modification of the software but that function was not used in this work. In short, each channel consists of a precision voltage-to-current transducer, analog switches for routing and creating (floating) negative voltages, as well as buffered Analog-to-Digital Converters for voltage and current samplings. The current command is generated through an eight-channel Digital-to-Analog Converter with an external voltage reference. Data logging and control were through serial communication between the computer and Teensy 4.0 Microcontroller Unit on the current source. The detailed block diagram is shown in fig. S1 and MATLAB Graphical User Interface in fig. S2. Complete bill of materials, schematics, and fabrication files at the time of publication are on Zenodo and will be updated on GitHub repository as we improve the design.

### Microscopy

All images were captured on an automated inverted microscope (Ti-2, Nikon) with motorized X-Y stage that was controlled using NIS-Elements (Nikon Instruments). The microscope was incubated at 37°C, and 5% CO_2_ was perfused into SCHEPHERD enclosure at a rate of 400 cubic centimeters per minute. The gas mixture was humidified through bubbler filled with sterile water to further reduce media evaporation during imaging. Fluorescence images were captured using a solid-state excitation source (Sola, Nikon) at 15% power and 4×/0.13 objective with an exposure of 400 ms to a complementary metal-oxide semiconductor camera (Qi-2, Nikon). A 10×/0.45 objective was used for individual cell tracking. Images were taken at 5-min interval. For actin imaging, a 40×/1.4 oil objective was used on a 24-well glass-bottom plate (P24-1.5H-N, CellVis). For cyst imaging, a 40×/1.25 silicone oil objective was used to provide extra working distance. A spinning disk confocal (X-light V3, CrestOptics) was used in conjunction with the microscope to obtain images under high magnification (Kinetix, Teledyne).

### Image analysis and quantification

All images were processed using FIJI (https://imagej.net/software/fiji), and MATLAB (MathWorks) was used for data visualization and statistical analysis. PIV of the tissue was performed using PIVLab (*85*), a MATLAB script for fast Fourier transform–based PIV. Analysis was performed over the entirety of the tissue, excluding the edge, with an initial pass of 64 by 64 pixels and then 32 by 32 pixels with 50% overlap. The results were filtered using the script’s velocity-based validation. Vectors beyond 7 SDs were replaced by interpolated vector from adjacent vectors. The filtered results were imported to MATLAB for processing. Resulting migration speed and directedness data are smoothed by using a moving average filter with a window size of 5.

Tracking was performed for 4′,6-diamidino-2-phenylindole (DAPI) –labeled images using TrackMate (*86*), an ImageJ plugin using simple Linear Assignment Problem (LAP) tracker. The results were filtered to exclude any incomplete track path and then were exported to an .XML file for MATLAB visualization. A custom script was used to calculate the average migration track.

For cell shape analysis in Fig. 4, each cell is masked with its outline to determine its aspect ratio and angle using analyze particle function in ImageJ. The results were plotted in GraphPad Prism 10.
